# Role of Mediterranean Diet Adherence on Endothelial Dysfunction in Autosomal Dominant Polycystic Kidney Disease Patients

**DOI:** 10.3390/biom16030447

**Published:** 2026-03-17

**Authors:** Luca Salomone, Danilo Menichelli, Vittoria Cammisotto, Valentina Castellani, Daniele Pastori, Pasquale Pignatelli, Anna Paola Mitterhofer, Francesca Tinti, Silvia Lai

**Affiliations:** 1 Nephrology Unit, Department of Translational and Precision Medicine, Sapienza University of Rome, 00185 Rome, Italy; luca.salomone@uniroma1.it (L.S.);; 2 Department of General Surgery, Surgical Specialties and Anesthesiology, Sapienza University of Rome, 00185 Rome, Italy; 3 Department of Medical and Cardiovascular Sciences, Sapienza University of Rome, 00185 Rome, Italy; 4 IRCCS Neuromed, 86077 Pozzilli, Italy; 5 Nephrology Unit, Department of Systems Medicine, Tor Vergata University of Rome, 00133 Rome, Italy

**Keywords:** Autosomal Dominant Polycystic Kidney Disease, ADPKD, Med-diet, endothelial dysfunction

## Abstract

Autosomal dominant polycystic kidney disease (ADPKD) is a genetic disorder characterized by progressive kidney enlargement by cyst formation. Endothelial dysfunction significantly contributes to chronic kidney disease (CKD). The Mediterranean diet (Med-diet) may reduce endothelial dysfunction in ADPKD patients, but its effect was not investigated in these patients. Our aim was to assess the relationship between Med-diet adherence and endothelial function biomarkers such as nitric oxide (NO) and endothelin-1 (ET-1). We enrolled ADPKD patients aged 18–70 years with CKD stages G2–G4. Adherence to the Med-diet was evaluated using the PREDIMED questionnaire. NO and ET-1 were evaluated at enrolment. Correlations and associations between these markers and Med-diet adherence were analysed. We enrolled 63 patients with ADPKD (mean age was 50.0 ± 11.8 years, 66.7% were female). A low/intermediate adherence to Med-Diet was assessed in 47 (74.6%) patients. When comparing patients with low/intermediate and high adherence, we found a higher NO and lower ET-1 serum concentration (*p* < 0.001 and *p* = 0.014, respectively) in patients with high adherence compared with low/intermediate ones. We found a significant correlation between Med-Diet adherence and NO (Spearman’s rs = 0.696, *p* < 0.001, 95%CI 0.542 to 0.805) and ET-1 serum concentrations (rs = −0.387, *p* = 0.002, 95%CI −0.579 to −0.154). For the univariable and multivariable linear regression analyses, we found an association between Med-Diet and NO (B: 0.547, 95%CI 0.050 to 0.121, *p* < 0.001) between Med-Diet and ET-1 (B: −0.327, 95%CI −0.157 to −0.020, *p* = 0.012). In conclusion, higher Med-Diet adherence seems to be associated with more favourable endothelial function in ADPKD patients.

## 1. Introduction

Autosomal Dominant Polycystic Kidney Disease (ADPKD) is the most common inherited kidney disorder and represents a major cause of chronic kidney disease (CKD) and end-stage kidney disease; the overall prevalence ranges from approximately ~1:1000 to 1:2000, with variability related to the diagnostic criteria, access to screening, and genetic heterogeneity [1,2]. ADPKD is burdened by several extra-renal complications, particularly an increased risk of cardiovascular events, which are among the leading causes of death in affected patients [3,4].

The most recent KDIGO guidelines for ADPKD emphasize, in addition to the use of disease-modifying drugs such as tolvaptan, an integrated approach that, in addition to pharmacological therapies, includes the management of risk factors and lifestyle habits (e.g., blood pressure control, dietary sodium restriction, physical activity, and behavioural counselling), with the aim of reducing cardiovascular complications and preserving kidney function [2].

In this context, endothelial dysfunction has emerged as a potential pathogenic and prognostic player. The endothelium regulates the vascular tone, inflammation, haemostasis, and arterial remodelling; its impairment—characterized by reduced bioavailability of nitric oxide (NO) and increased vasoconstrictor mediators such as endothelin-1 (ET-1)—is an early determinant of atherosclerosis and heightened cardiovascular risk [5,6,7].

At the molecular level, endothelial dysfunction reflects a maladaptive shift in the tightly regulated ET-1/NO axis toward vasoconstriction, oxidative stress, and pro-inflammatory signalling. ET-1 is generated from prepro-ET-1 via sequential processing to “big endothelin” and final cleavage by endothelin-converting enzymes (ECEs), resulting in sustained paracrine/autocrine activation of G-protein-coupled endothelin receptors [8,9]. Signalling through ETA receptors—predominantly expressed on vascular smooth muscle—promotes vasoconstriction and vascular remodelling via calcium-dependent pathways and RhoA/ROCK, and it can amplify redox stress by stimulating NADPH oxidase-dependent superoxide production, which directly quenches NO and propagates redox-sensitive inflammatory/proliferative cascades [8,10]. Conversely, endothelial ETB receptors exert counter-regulatory actions by stimulating NO (and prostacyclin) release and by functioning as “clearance receptors” that remove circulating ET-1; disruption of this protective ETB arm can therefore increase the ET-1 bioavailability and further bias vascular tone toward constriction [8,11]. On the vasodilator side, NO—mainly produced by endothelial nitric oxide synthase (eNOS) in response to shear stress and kinase-dependent phosphorylation—signals through soluble guanylate cyclase and cGMP to induce vasorelaxation and confer anti-platelet, anti-inflammatory, and anti-proliferative effects [12]. Importantly, oxidative stress not only reduces NO bioavailability through rapid reaction with superoxide (forming peroxynitrite) but also favours eNOS “uncoupling” (e.g., via tetrahydrobiopterin oxidation), converting eNOS into a net source of reactive oxygen species and reinforcing a feed-forward cycle of nitro-oxidative stress and endothelial dysfunction [12,13]. This bidirectional crosstalk is clinically relevant because NO can physiologically restrain ET-1 synthesis and signalling, whereas ET-1-driven oxidative pathways can suppress NO-mediated vasoprotection, thereby stabilizing a vasoconstrictor, pro-atherogenic phenotype [14].

In ADPKD, endothelial dysfunction may also occur in early stages, before a marked decline in estimated glomerular filtration rate (eGFR), suggesting that it may contribute to renal damage and disease progression rather than being merely a consequence of CKD-related “secondary” effects [5,6]. Experimental and clinical evidence supports a role for polycystins (PKD1/PKD2) in maintaining vascular homeostasis: PKD1/2 mutations, in addition to causing cyst formation, appear to be responsible for endothelial alterations such as reduced NO-dependent vasodilation and arterial hypertension [4,7]. Moreover, oxidative stress and inflammation—common in CKD and documented in ADPKD—may inactivate NO and amplify vasoconstrictor signalling, creating a vicious cycle between endothelial dysfunction, hypertension, and progression of end-organ damage [6].

Diet is a modifiable determinant of vascular health. The Mediterranean Diet (Med-Diet) is associated with reduced cardiovascular risk and improvements in intermediate measures of endothelial function [15,16]. In a meta-analysis of randomized trials, Med-Diet-based interventions showed a significant increase in endothelial function as assessed by flow-mediated dilation, indicating a beneficial effect already in the early phases of the atherosclerotic process [16]. Studies within the PREDIMED program and related analyses further suggest that Mediterranean dietary patterns, especially when enriched with extra-virgin olive oil or nuts, may modulate pathways involved in blood pressure regulation and endothelial biology, including biomarkers related to NO and ET-1 [17,18]. From a nephrology perspective, despite methodological heterogeneity, systematic reviews and prospective studies indicate an association between greater adherence to the Med-Diet and a lower risk of CKD and/or slower decline in kidney function in populations at high cardiometabolic risk [19,20]. However, ADPKD-specific data—particularly regarding the potential benefits of the Med-Diet on endothelial function—remain limited, despite biological plausibility and the clinical relevance of cardiovascular risk in this population. For this reason, we want to investigate the role of the Med-Diet on the endothelial function in patients with ADPKD.

## 2. Methods

### 2.1. Study Design and Subjects

We performed an observational, monocentric study on 63 consecutive ADPKD patients at the University Hospital “Policlinico Umberto I” of Rome, Sapienza University of Rome, Italy. Patients have been enrolled from June to November 2024. The study was approved by the local ethics committee of Sapienza University (No 7669 Prot. 0809/2024) in 11 September 2024 and was conducted according to the 1975 Declaration of Helsinki. All patients signed informed written consent at study entry.

### 2.2. Inclusion Criteria

Patients aged 18–70 years with ADPKD (defined according to the Pei’s criteria [21]) and G2 to G4 stages of CKD (15 mL/min/1.73 m^2^ ≤ eGFR ≤ 90 mL/min/1.73 m^2^), calculated using the CKD-EPI equation according to Kidney Disease Improving Global Outcomes (KDIGO) guidelines, were recruited [22].

### 2.3. Exclusion Criteria

Patients with an age over 70 years, or CKD stage G5 or renal replacement therapy were excluded from this study. Furthermore, we excluded also patients with renal transplant recipients and patients with a clinical indication to follow a specific diet aimed at delaying CKD progression (e.g., low-protein or very low-protein diets, use of ketoanalogues). In addition, we excluded all patients with autoimmune and chronic inflammatory diseases that could cause worsening renal function in patients with APDKD. Moreover, patients that refused to give consent and with missing data were also excluded.

### 2.4. Clinical and Laboratory Measurement

During the initial clinical examination, a comprehensive medical history was obtained, including comorbidities and cardiovascular and renal risk factors such as arterial hypertension, diabetes mellitus, smoking habits, obesity status (defined according to body mass index [BMI], evaluated by weight (kg)/[height (m)]^2^). Routine blood tests were performed, including a complete blood count, creatinine, urea, uric acid, and serum electrolytes (sodium, potassium, calcium, phosphorus). Additionally, patients’ current pharmacological treatments were documented (angiotensin converting enzyme inhibitors/angiotensin [ACE-I/ARBs], receptor blockers, beta blockers, calcium channel blockers [CCBs], diuretics, levotiroxin, allopurinol, tolvaptan and octreotide).

### 2.5. Endothelial Dysfunction Assessment

Peripheral venous blood samples were collected into tubes containing 3.8% sodium citrate anticoagulant or without additives. Samples were processed by centrifugation at 300× *g* for 10 min at room temperature to separate plasma and serum. Aliquots were then stored at −80 °C for 6 months from collection, pending analysis.

### 2.6. Endothelin-1 (ET-1)

Serum concentrations of ET-1 were quantified by enzyme-linked immunosorbent assay (ELISA) utilizing a commercially available kit (TEMA Ricerca srl, Castenaso, Bologna, Italy), following the manufacturer’s instructions. Results are expressed in pg/mL. The intra- and inter-assay coefficients of variation were consistently below 10%.

### 2.7. Nitric Oxide (NO)

Due to the intrinsic instability and brief half-life of nitric oxide (NO)—approximately 6 s—its measurement was indirectly performed by quantifying stable metabolic by-products, namely, nitrite (NO_2_^−^) and nitrate (NO_3_^−^), collectively referred to as NOx [23]. NOx levels were assessed in serum samples using a colorimetric assay kit (Abcam, Cambridge, UK). A volume of 100 µL of sample was incubated under constant stirring at 37 °C for 10 min. Concentrations were expressed in µM. The intra- and inter-assay coefficients of variation were 2.9% and 1.7%, respectively.

### 2.8. Med-Diet Definition and Classification

The Med-Diet emphasizes foods commonly consumed in Mediterranean countries, favouring cereals, fruits, vegetables, seeds, and olive oil while limiting red meats and animal fats. It also includes moderate consumption of fish, poultry, legumes, eggs, dairy, red wine, and sweets. At enrolment, patients completed the PREDIMED [17] questionnaire to assess adherence to the Med-Diet. The PREDIMED questionnaire is a simple and practical tool designed to assess how closely a person follows the eating habits typical of the Med-Diet. The questionnaire consists of 14 questions, each focusing on a specific aspect of the Med-Diet. For example, it investigates the use of olive oil as the main source of fat in cooking, the daily intake of fruits, vegetables, and legumes, and the frequency of consuming fish, nuts, and whole grains. It also considers moderate consumption of red wine during meals, a characteristic element of the Mediterranean tradition. At the same time, it evaluates the limitation of less healthy foods such as red meat, processed meats, sweets, and sugary drinks. Each response is worth 1 point if the dietary habit aligns with Med-Diet criteria, or 0 points if it does not. The total score ranges from 0 to 14, allowing for classification of adherence to the diet as follows: 0–5 indicates low adherence, 6–9 moderate adherence, 10–14 high adherence.

Thanks to its simplicity, the PREDIMED questionnaire is highly useful in both clinical practice and research. It enables the rapid identification of dietary habits that could be improved and provides a foundation for correlating adherence to the Med-Diet with various health indicators, such as oxidative stress regulation, cardiovascular disease prevention, and the management of chronic conditions like kidney disease [24,25,26].

### 2.9. Study Endpoints

The study endpoints were the evaluation of endothelial dysfunction biomarkers such as ET-1 and NO and their associations with different Med-Diet adherence levels.

### 2.10. Sample Size Calculation

Estimating a 35% increase in NO serum levels according to a previous study [27], with a statistical power of 80% and alpha error of 0.05, the required sample size was 50 patients.

### 2.11. Statistical Analysis

Categorical variables were reported as numbers and percentages, which were compared using Pearson’s χ2 test. The mean and standard deviation (SD) or median and interquartile range (IQR) were used for continuous variables, which were compared by Student’s t-test or the Mann–Whitney U test, respectively. Normal distribution of variables was checked by the Kolmogorov-Smirnov test. We used Student’s unpaired t-test and Pearson’s product-moment correlation analysis to evaluate normally distributed continuous variables and an appropriate nonparametric test (Mann–Whitney U test and Spearman’s rank correlation test) for the other variables. Group comparisons were performed using Fisher’s F-test (ANOVA) or the Kruskal–Wallis test when needed.

Descriptive analysis according to Med-Diet adherence (low/intermediate vs high adherence) describing clinical and laboratory characteristics were performed. We evaluated the correlation between the Med-Diet and endothelial dysfunction markers using the Spearman rank correlation test. We then performed univariable and multivariable stepwise linear regression analysis adjusted for potential confounders (age, sex and eGFR) to evaluate the association between the Med-Diet and endothelial dysfunction markers.

All tests were 2-tailed and only *p*-values < 0.05 were considered statistically significant. The analyses were performed using SPSS 25.0 software (IBM, Armonk, NY, USA) and MedCalc (18.2.1).

## 3. Results

### 3.1. Baseline Characteristics

Overall, we enrolled 63 consecutive patients with ADPKD, the mean age was 50.0 ± 11.8 years and 66.7% were female (Table 1). Several patients were affected by hypertension (82.5%) and anaemia (17.7%). A low/intermediate adherence to Med-Diet was identified in 47 (74.6%) patients.

When comparing patients with low/intermediate adherence and high adherence, we found no statistical differences regarding clinical risk factors (Table 1) such as arterial hypertension; smoking habits; anaemia; diabetes; and obesity, expressed as BMI (Table 1). No difference between the two groups was found regarding the drugs administered (Table 1). Among the laboratory findings, we found a higher eGFR without significant differences in electrolytes between the two groups (Table 1).

**Figure 1:**
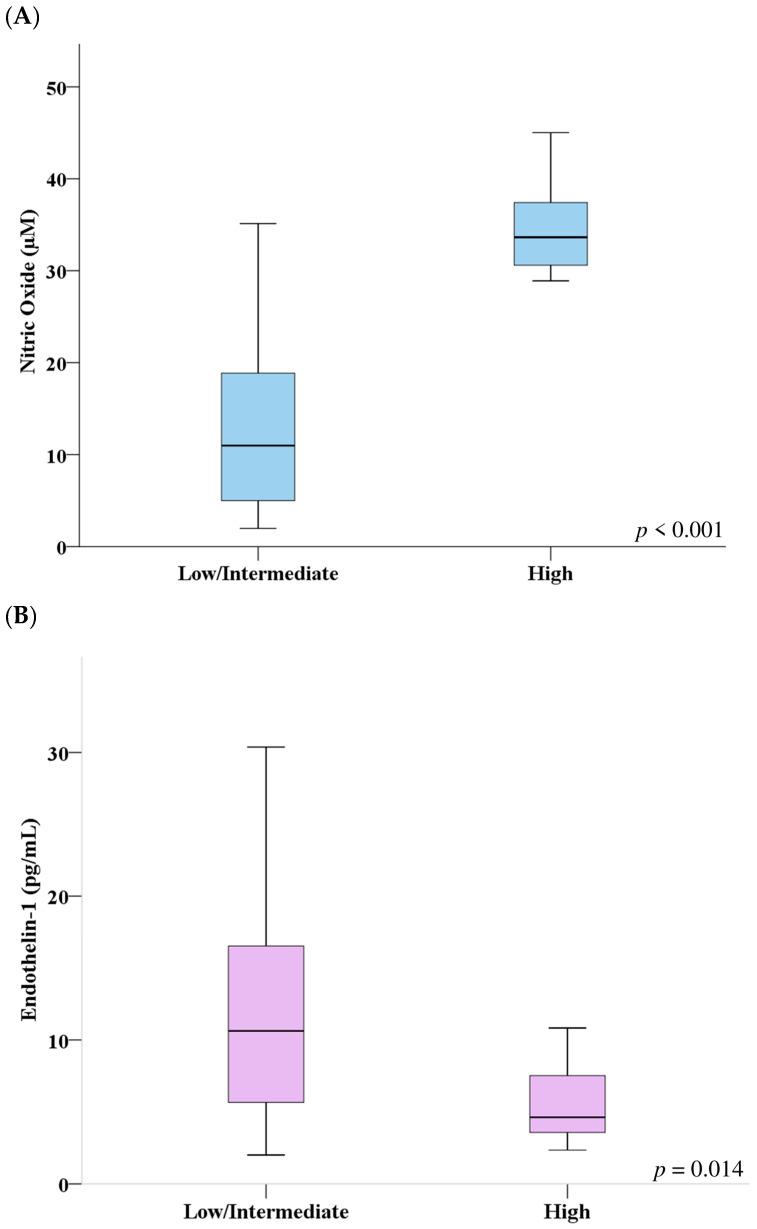
Comparison of serum concentrations of nitric oxide (**A**) and endothelin-1 (**B**) between different Mediterranean Diet adherence levels.

When evaluating endothelial dysfunction biomarkers, we found significantly higher NO and lower ET-1 serum concentrations (*p* < 0.001 and *p* = 0.014, respectively) in patients with high adherence compared with low/intermediate adherence ones (Figure 1).

### 3.2. Med-Diet and Endothelial Dysfunction

**Figure 2:**
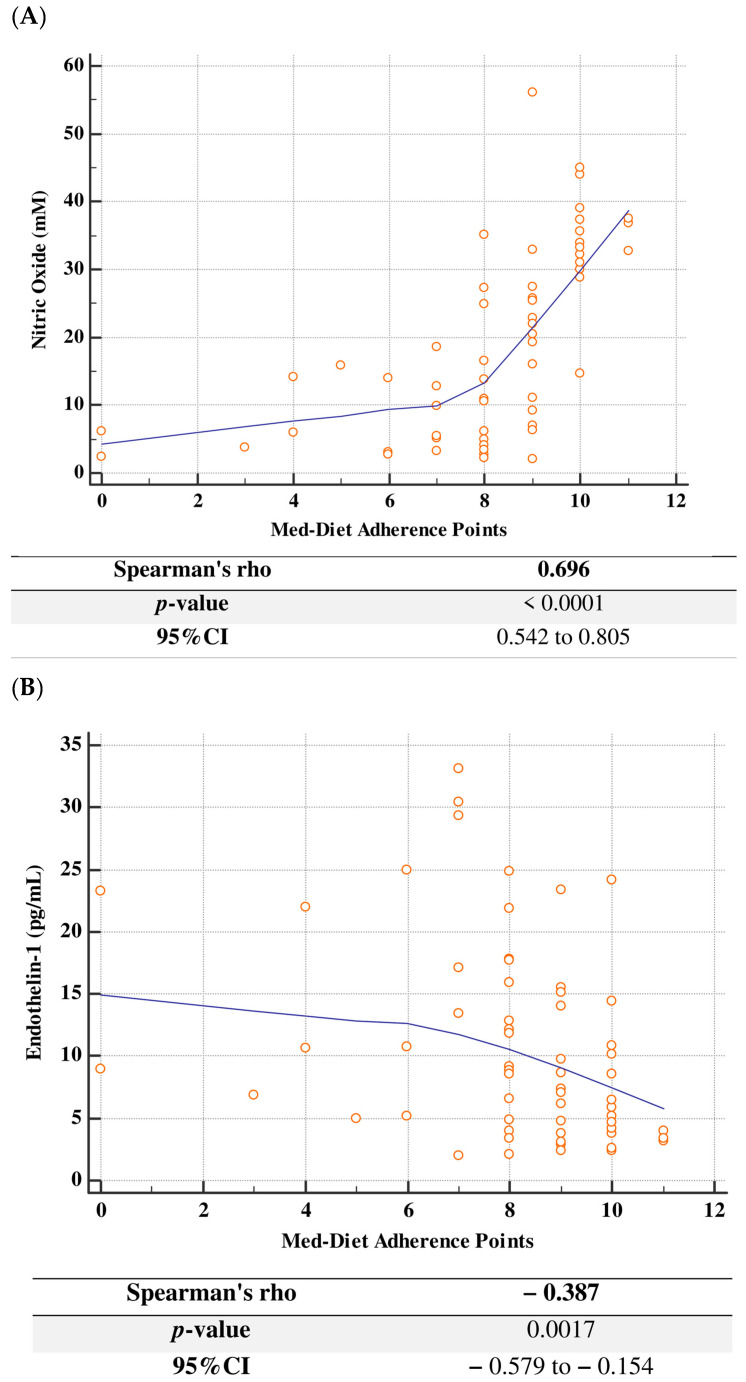
Correlations between Mediterranean Diet adherence and nitric oxide (**A**) and endothelin-1 (**B**). CI: confidence interval, Med-Diet: Mediterranean diet.

Spearman’s correlation analysis showed a significant correlation between the Med-Diet adherence points and NO serum concentration (r_s_ = 0.696, *p* < 0.001, 95%CI 0.542 to 0.805) (Figure 2A) and the Med-Diet adherence points and ET-1serum concentration (r_s_ = −0.387, *p* = 0.002, 95%CI −0.579 to −0.154) (Figure 2B).

We then performed univariable and multivariable stepwise linear regression analyses to assess the effect of Med-Diet adherence on NO and ET-1 serum concentrations.

The univariable analysis found that the Med-Diet was associated with a higher NO serum concentration (B: 0.568, 95%CI 0.059 to 0.128, *p* < 0.001), as shown in Table 2, Panel A, and with a lower ET-1 serum concentration (B −0.305, 95%CI −0.154 to −0.017, *p* = 0.015), as shown in Table 2, Panel B.

From the multivariable stepwise linear regression analysis, the associations between Med-Diet adherence and NO (B: 0.547, 95%CI 0.050 to 0.121, *p* < 0.001) and ET-1 (B: −0.327, 95%CI −0.157 to −0.020, *p* = 0.012) were confirmed, as shown in Table 2, Panels A and B respectively.

When performing univariable and multivariable linear regression analyses comparing high vs low/intermediate Med-Diet adherence, we found a significant direct association between the high Med-Diet adherence group and NO (Table 3, Panel A) and a significant inverse association between the high Med-Diet adherence group and ET-1 (Table 3, Panel B).

## 4. Discussion

In this single-centre observational study of 63 consecutive patients with ADPKD (enrolled between June and November 2024), Med-Diet adherence assessed using the PREDIMED questionnaire was associated with a more favourable endothelial biomarker profile. Specifically, participants with high adherence (*n* = 16) showed higher serum NO levels and lower endothelin-1 (ET-1) levels than those with low/intermediate adherence (*n* = 47), with statistically significant differences.

Consistently, Med-Diet adherence correlated positively with NO and inversely with ET-1, and these associations remained evident in stepwise regression models that included age, sex, and eGFR.

These findings are plausible within the pathophysiological context of ADPKD, in which endothelial dysfunction and impaired NO bioavailability have been described, even at relatively early stages, contributing to the characteristic vascular phenotype (hypertension, arterial stiffness, reduced endothelium-dependent vasodilation) [7,28]. In this framework, the endothelin pathway is of particular interest: increased ET-1 levels have been reported in ADPKD cohorts and have been associated with hypertension and less favourable renal/overall prognosis [29,30]. Therefore, the observation that lower ET-1 levels appear to be associated with greater adherence to the Med-Diet in our sample may be interpreted as a signal of “alignment” between a cardioprotective dietary pattern and a less vasoconstrictive vascular milieu, without implying directionality or causality.

Comparison with the nutritional and cardiovascular literature further supports the biological coherence of these findings. A meta-analysis of randomized trials reported that Med-Diet-based interventions are accompanied by improved endothelial function as assessed by flow-mediated dilation (FMD), a widely validated functional endpoint [16]. Moreover, PREDIMED-based analyses have described changes in endothelial markers involved in blood pressure regulation, including NO and ET-1, among participants assigned to Med-Diet variants enriched with extra-virgin olive oil or nuts [31]. Our results extend this line of evidence to an ADPKD population, in which disease-specific data on the Med-Diet and endothelial biomarkers remain limited, yet clinically relevant given the high cardiovascular burden [32].

Interpretation of biomarkers nonetheless requires caution, particularly for NO. In our protocol, NO was measured indirectly as nitrite/nitrate (NO) due to NO instability; this approach is common, but the literature emphasizes that NO may reflect different components (endogenous production, diet, metabolism), and that the nitrite fraction is more closely linked to eNOS activity than nitrate [23,33]. Consequently, the observed association between the PREDIMED score and NO may also be influenced by dietary nitrate intake (e.g., leafy green vegetables), in addition to differences in endothelial bioactivity; this should be considered a potential source of residual variability that was not fully controlled [23,33].

A central potential confounder is kidney function: the high-adherence group had a higher eGFR; therefore, it might appear that the group with greater adherence to the Med-Diet has a slower rate of kidney disease progression. However, this statistically significant finding could be affected by differences in disease severity or by clustering of unmeasured healthy behaviours; moreover, reverse causation cannot be excluded, whereby patients with more advanced CKD adopt different dietary patterns or experience dietary constraints not fully captured by the PREDIMED questionnaire, which is a brief tool administered at a single time point [17].

Importantly, the association between adherence and biomarkers remained present in stepwise models that included age, sex, and eGFR. From a clinical and therapeutic standpoint, many variables and treatments did not differ between the groups (including ACE inhibitors/ARBs, beta-blockers, calcium channel blockers, and diuretics), reducing the likelihood that the findings are explained solely by major pharmacological differences, although residual confounding remains possible.

Overall, these results suggest that in patients with ADPKD, greater adherence to the Med-Diet appears to be associated with a combination of higher NO and lower ET-1, consistent with external evidence linking Mediterranean dietary patterns to better “endothelial health”, both functionally (FMD) and at the biomolecular level (NO/ET-1) [7,16,31]. The role of endothelial dysfunction is well known in CKD, and several drugs, such as atrasentan, have been evaluated to block endothelin receptors and improve albuminuria and kidney function [34]. In this context, a high adherence to the Med-Diet may represent a potential low-cost and high-effectiveness adjuvant of pharmacological treatment, reducing ET-1 serum levels, as shown by our preliminary results. From a research perspective, these findings support the need for prospective and/or interventional studies in ADPKD that integrate functional assessments (FMD, PWV), a more granular and repeatedly administered dietary evaluation over time, and broader control of behavioural confounders (physical activity, sleep, stress, sodium and nitrate intake) to clarify the robustness and directionality of the observed associations [16,23,30]. In ADPKD, endothelial dysfunction appears to be a key process closely associated with disease progression. In this setting, reduced FMD, as a marker of impaired endothelium-dependent vasodilation, is likely linked to increased vasoconstrictive and pro-fibrotic activity mediated by ET-1. Kocyigit et al. showed that higher endothelin-1 levels are associated with more advanced CKD stages, the presence of hypertension, and poorer renal survival. Overall, reduced FMD and increased ET-1 seem to represent two complementary aspects of the same vascular injury process: on the one hand, the loss of protective endothelial function, and on the other, the activation of vasoconstrictive, ischemic, and fibrotic pathways that promote ADPKD progression [29].

A key aspect of these findings is their alignment with a growing body of evidence suggesting that endothelial dysfunction is an early and potentially intrinsic feature of ADPKD. In humans, artery-resistance studies and clinical investigations have reported impaired NO-mediated vasodilation in ADPKD, supporting reduced endothelial NO bioactivity as a plausible contributor to the hypertensive and pro-atherogenic vascular phenotype [15,35,36]. Complementary experimental data indicate that endothelial polycystin deficiency can promote endothelial dysfunction and hypertension, reinforcing biological plausibility for a disease-linked disruption of endothelial signalling pathways that regulate vascular tone [15].

From a mechanistic nutrition standpoint, several components of a Mediterranean dietary pattern could converge on the ET-1/NO axis. A higher intake of nitrate-rich vegetables can support the nitrate–nitrite–NO pathway and contribute to NO bioactivity, potentially amplifying NO signalling even when eNOS-dependent production is impaired by oxidative stress [37,38]. In parallel, Mediterranean patterns are enriched in polyphenols and antioxidant micronutrients that can modulate endothelial oxidative stress and NOS regulation; in Mediterranean diet cohorts, polyphenol exposure has been associated with increases in plasma NO-related measures and favourable blood pressure profiles [39,40]. These parallel pathways provide a coherent mechanistic rationale for the association of higher PREDIMED scores with higher NOx and lower ET-1 while remaining compatible with the non-causal, cross-sectional design of the present study.

In addition, the therapeutic context underscores the translational interest of endothelin and NO in CKD. Endothelin receptor antagonism has been pursued as a strategy to improve renal outcomes, and in the SONAR trial, atrasentan reduced renal events in a selected high-risk CKD population, albeit with class-related safety considerations, such as fluid retention [41]. Although not ADPKD-specific, these data emphasize that endothelin biology is a modifiable pathway, strengthening the rationale to investigate low-risk, lifestyle-based strategies that may shift the endothelin/NO balance in a favourable direction, particularly in ADPKD, where cardiovascular risk is substantial.

Finally, in our cohort of patients with ADPKD, greater adherence to the Med-Diet was associated with a potentially more favourable endothelial profile, with lower endothelin-1 (ET-1) levels and higher NO values. This observation is consistent, on the one hand, with evidence describing endothelial dysfunction and alterations in NO-related pathways in ADPKD [5] and, on the other hand, with randomized evidence and Mediterranean-diet-based analyses showing improved endothelial function and modulation of NO/ET-1 biomarkers in relation to Mediterranean dietary patterns [16,31]. A potential direction suggested by these findings is the use of nutritional therapy as a component of conservative ADPKD management. Overall, the results support considering nutritional counselling as part of an integrated approach to ADPKD care, in line with KDIGO recommendations that emphasize lifestyle interventions alongside disease-specific therapies [2], while dedicated prospective and interventional studies are still needed to confirm the robustness and clinical relevance of these associations. the association between Med-Diet adherence and a higher-NO/lower-ET-1 biomarker profile is biologically coherent with contemporary concepts of early ADPKD vasculopathy and with mechanistic links between diet, NO bioactivity, oxidative stress, and endothelin signalling [15,35,36,37,39].

### Limitations

Among the main limitations of the present study are its observational, single-centre, and cross-sectional nature, which do not allow for directionality or causal relationships between Med-Diet adherence and endothelial biomarkers to be established. The relatively small sample size and imbalance between the groups (high adherence being a minority) may reduce the statistical power and generalizability of the findings. However, the small sample size reflected the real-world enrolment of patients with ADPKD due to low prevalence of the disease, estimated by 1 in 1000 based on PKD1 and PKD2 protein-truncating mutations by population whole exome or genome sequencing and 4 in 10,000 persons on surveys performed on national Western registries [42]. Dietary assessment using the PREDIMED questionnaire at a single time point may not accurately reflect long-term dietary patterns and may lead to misclassification. Although we performed a multivariable linear regression analysis adjusting for eGFR, we could not exclude that it could remain as a potential confounding factor and limitation of our results. Finally, despite adjustments for age, sex, and eGFR, the possibility of residual confounding (physical activity, stress, sleep, socioeconomic status, sodium/nitrate intake) and reverse causation remains, particularly in light of the differences in eGFR between groups.

Additional limitations should be acknowledged. NO was measured as NOx, which reflects both endothelial NO generation and dietary nitrate-derived metabolites; therefore, differences in vegetable intake and nitrate exposure may contribute to NOx variability and could partially mediate (or confound) the observed association with PREDIMED score [37,38]. Moreover, we did not include functional vascular endpoints (e.g., FMD or pulse wave velocity), which would help to connect biomarker changes to vascular physiology [43].

As future perspective, further studies investigating ET-1, NO and their association with functional vascular assessment and biomarker as Hypoxia-Inducible Factor 1-α (HIF-α) levels could provide additional insight into the potential role of renal hypoxia as a consequence of endothelial dysfunction and help to further clarify the pathophysiological mechanisms underlying ADPKD progression.

## 5. Conclusions

In conclusion, higher adherence to Med-Diet seems to be associated with more favourable endothelial function serum biomarkers in ADPKD patients. Future prospective studies should combine repeated dietary assessment with functional vascular testing and broader biomarker panels to strengthen the causal inference and to define the clinical relevance of these pathways in ADPKD.

## Figures and Tables

**Table 1:** Clinical characteristics according to Mediterranean Diet adherence. ACE-I/ARBs: angiotensin converting enzyme inhibitors/angiotensin receptor blockers, BMI: body mass index, CCB: calcium channel blockers, COPD: chronic obstructive pulmonary disease, eGFR: estimated glomerular filtration rate, ET-1: endothelin-1, NO: nitric oxide, PTH: parathyroid hormone. * median [interquartile range].

	Total Cohort (*n*: 63)	Low/Intermediate Adherence (*n*: 47)	High Adherence (*n*: 16)	*p*-Value
**Age (years)**	50.0 ± 11.8	50.1 ± 11.7	49.8 ± 12.5	0.937
**Female sex (%)**	42 (66.7)	29 (61.7)	13 (81.3)	0.152
**Hypertension (%)**	52 (82.5)	39 (83.0)	13 (81.3)	0.875
**Smoking habits (%)**	8 (12.7)	6 (12.8)	2 (12.5)	0.978
**BMI**	24.4 ± 4.5	24.1 ± 4.2	25.4 ± 5.2	0.319
**Obesity (%)**	9 (14.3)	6 (12.8)	3 (18.8)	0.555
**Dyslipidaemia (%)**	8 (12.7)	6 (12.8)	2 (12.5)	0.978
**Previous cerebrovascular disease (%)**	0 (0.0)	0 (0.0)	0 (0.0)	-
**Previous cardiovascular disease (%)**	2 (3.2)	2 (4.3)	0 (0.0)	0.402
**COPD (%)**	0 (0.0)	0 (0.0)	0 (0.0)	-
**Heart failure (%)**	0 (0.0)	0 (0.0)	0 (0.0)	-
**Anaemia (%)**	11 (17.7)	9 (19.6)	2 (12.5)	0.524
**Diabetes (%)**	1 (1.6)	1 (2.1)	0 (0.0)	0.556
**Therapy**				
**ACE-I/ARBs (%)**	47 (78.3)	34 (77.3)	13 (81.3)	0.741
**Beta blockers (%)**	9 (15.3)	7 (16.3)	2 (12.5)	0.720
**CCB (%)**	27 (45.8)	21 (47.7)	6 (40.0)	0.604
**Diuretics (%)**	6 (9.8)	5 (11.1)	1 (6.3)	0.575
**Levotiroxin (%)**	7 (11.5)	4 (8.9)	13 (8.8)	0.288
**Tolvaptan/octreotide (%)**	14 (23.0)	13 (28.9)	1 (6.3)	0.060
**Allopurinol (%)**	21 (35.0)	18 (40.9)	3 (18.8)	0.112
**Statins (%)**	8 (12.7)	6 (12.8)	2 (12.5)	0.978
**Blood laboratory findings**				
**Haemoglobin (g/dL)**	13.0 ± 1.5	12.9 ± 1.5	13.3 ± 1.4	0.326
**Platelets (×10^3^/µL)**	214.0 [182.0, 272.5]	213.5 [182.0, 268.8]	214.0 [159.0, 280.0]	0.971
**Calcium (mg/dL)**	9.3 ± 1.2	9.2 ± 1.4	9.5 ± 0.5	0.481
**Phosphorus (mg/dL)**	3.8 ± 0.7	3.8 ± 0.7	3.6 ± 0.8	0.389
**Sodium (mmol/L)**	140.2 ± 3.3	140.5 ± 2.9	139.5 ± 4.2	0.305
**Potassium (mmol/L)**	4.8 ± 0.5	4.8 ± 0.5	4.6 ± 0.5	0.316
**Uric acid (mg/dL)**	6.0 [5.0, 6.8]	6.0 [5.2, 6.8]	6.1 [4.6, 6.9]	0.609
**Urea (mg/dL)**	90.2 ± 43.4	92.1 ± 43.2	84.9 ± 44.9	0.571
**Creatinine (mg/dL)**	1.6 ± 0.9	1.7 ± 0.8	1.24 ± 1.02	0.070
**eGFR (mL/min/1.73 m^2^)**	55.9 ± 28.9	49.8 ± 26.0	73.8 ± 30.4	0.003
NO (µM) *	16.0 [6.1, 30.1]	11.0 [4.9, 19.2]	33.6 [30.3, 37.4]	<0.001
ET-1 (pg/mL) *	8.9 [4.6, 15.1]	10.6 [5.2, 17.1]	4.9 [3.5, 9.8]	0.014

**Table 2:** Results of univariable and stepwise multivariable linear regressions to evaluate the associations of nitric oxide (Panel A) and endothelin-1 (Panel B) with Mediterranean diet adherence. * Stepwise model included age, sex and estimated glomerular filtration rate.

Panel A	Beta	95%CI		*p*-Value
***Univariable***	0.568	0.059	0.128	<0.001
Multivariable *	0.547	0.050	0.121	<0.001
**Panel B**	**Beta**	**95%CI**		***p*-Value**
***Univariable***	−0.305	−0.154	−0.017	0.015
Multivariable *	−0.327	−0.157	−0.020	0.012

**Table 3:** Results of univariable and stepwise multivariable linear regressions to evaluate the associations of high Med-Diet adherence (vs. low/intermediate adherence) with nitric oxide (Panel A) and endothelin-1 (Panel B). * Stepwise model included age, sex and estimated glomerular filtration rate.

Panel A	Beta	*p*-Value
***High vs. low/intermediate***	0.664	<0.001
***High vs. low/intermediate*** *	0.656	<0.001
**Panel B**	**Beta**	***p*** **-Value**
***High vs. low/intermediate***	−0.288	0.022
***High vs. low/intermediate*** *	−0.313	0.017

## Data Availability

Data available on request due to privacy restrictions. The data presented in this study are available on request from the corresponding author.
